# Metabolomics by UHPLC-Q-TOF Reveals Host Tree-Dependent Phytochemical Variation in *Viscum album* L.

**DOI:** 10.3390/plants10081726

**Published:** 2021-08-20

**Authors:** Tim Jäger, Carla Holandino, Michelle Nonato de Oliveira Melo, Evelyn Maribel Condori Peñaloza, Adriana Passos Oliveira, Rafael Garrett, Gaétan Glauser, Mirio Grazi, Hartmut Ramm, Konrad Urech, Stephan Baumgartner

**Affiliations:** 1 Society for Cancer Research, Hiscia Institute, Kirschweg 9, 4144 Arlesheim, Switzerland; lazic@mdpi.com (T.J.); m.grazi@vfk.ch (M.G.); h.ramm@vfk.ch (H.R.); k.urech@vfk.ch (K.U.); 2 Center for Complementary Medicine, Institute for Infection Prevention and Hospital Epidemiology, Faculty of Medicine, University of Freiburg, Breisacher Str. 115b, 79106 Freiburg, Germany; 3 Institute of Integrative Medicine, University of Witten/Herdecke, Gerhard-Kienle-Weg 4, 58313 Herdecke, Germany; 4 Laboratório Multidisciplinar de Ciências Farmacêuticas, Pharmacy College, Federal University of Rio de Janeiro, Rio de Janeiro 21941-902, Brazil; michellenonato.far@gmail.com (M.N.d.O.M.); evelyn.condoloza@gmail.com (E.M.C.P.); passosoliv@hotmail.com (A.P.O.); 5 Metabolomics Laboratory, Chemistry Institute, Federal University of Rio de Janeiro, Rio de Janeiro 21941-598, Brazil; rafael_garrett@iq.ufrj.br; 6 Neuchatel Platform of Analytical Chemistry, University of Neuchâtel, Avenue de Bellevaux 51, 2000 Neuchâtel, Switzerland; gaetan.glauser@unine.ch; 7 Institute of Complementary and Integrative Medicine, University of Bern, Freiburgstrasse 46, 3010 Bern, Switzerland

**Keywords:** *Viscum album*, mistletoe, host tree, metabolite fingerprinting, multivariate analysis

## Abstract

*Viscum album* L., commonly known as European mistletoe, is a hemi-parasitic plant of the Santalaceae family. The in vitro and in vivo effects of *V. album* differ, according to its host tree. However, little is known about the host-dependent phytochemical diversity in *V. album*. In this study, the metabolic profiles of *V. album* ssp. *album* from *Malus domestica* Bork., *Quercus robur* L., and *Ulmus carpinifolia* Gled were compared. Leaves, stems, and berries were collected in Switzerland, by the same procedure, in September 2016 and 2017. The methanolic extracts were analyzed by ultra-performance liquid chromatography, coupled to electrospray quadrupole time-of-flight mass spectrometry in positive ionization mode. The data were submitted to partial-least square discriminant analysis (PLS-DA) and the results showed that the *V. album* ssp. *album* samples were clustered into three groups, according to the three distinct host trees. Seven compounds, with high VIP scores (variable importance in projection), were responsible for this differentiation. The following four compounds were detected in both the harvest years: arginine, pipecolic acid or lysine, dimethoxycoumarin, and sinapyl alcohol, suggesting their use as host specific *V. album* biomarkers. The present work highlights the importance of standardized harvest and analytical procedures for the reproducibility of the chemical results of herbal materials.

## 1. Introduction

*V. album* L. (Santalaceae), popularly known as mistletoe, is an ancient medicinal plant used by European and Asian communities. Its ethnomedicinal use includes the treatment of epilepsy, anxiety, hypertension, internal bleeding, and atherosclerosis [1,2]. The remarkable interest in this plant arose in 1920, when mistletoe was introduced as an anticancer agent by Steiner and Wegman [3]. Its antitumor activity is mainly attributed to viscotoxins and lectins, which are high-molecular-weight compounds [4]. Nowadays, it is used as a complementary therapy for the treatment of different types of cancer, such as breast, colorectal, and pancreatic cancer, to prolong survival and to increase the quality of life [5,6]. Other studies have shown *V. album*’s pharmacological potential as anti-inflammatory [7], antihepatotoxic [8], hypoglycemic and antioxidant [9,10], antimicrobial [11], antiepileptic, sedative, and antipsychotic, as well as for cardiac diseases [1,2].

*V. album* ssp. *album* can adapt to different conditions, being able to grow on different deciduous host trees, including *Malus domestica* Bork., *Quercus robur* L., and *Ulmus carpinifolia* Gled. The mistletoe plant morphology shows a dense and rounded aspect, with leaves in abundance and ripe white berries in the European winter [12]. In contrast to other traditional medicinal plants, *V. album* is a hemi-parasite species, since its growth occurs by the absorbance of water, sugars, amino acids, and minerals from the host tree; however, it is also able to produce primary and secondary metabolites. This parasitism occurs through a root system called haustorium, which establishes connections with the host tree xylem [12]. However, little is known about the host-depending chemical differences of *V. album*, as well as the metabolome influence on the biological activity of mistletoe preparations [13,14].

Plant metabolomics has become a potent experimental strategy, since it allows the simultaneous evaluation of many metabolites under different conditions, providing a rapid and reliable picture of the plant chemical content [15,16,17]. The metabolome analysis is mainly based on nuclear magnetic resonance (NMR) and liquid chromatography coupled to mass spectrometry (LC-MS) methodologies. A vast number of small molecules, derived from primary and secondary *Viscum album* metabolism, was previously described in mistletoe-fermented aqueous extracts, highlighting the sensitivity and selectivity of LC-MS for metabolite detection in complex matrices [18]. 

In the present study, a UHPLC-QTOF-MS untargeted metabolomic approach was used, to explore the metabolic composition of *Viscum album* ssp. *album*, grown on the following three different deciduous host trees: *M. domestica*, *Q. robur*, and *U. carpinifolia*. Moreover, the identification of specific biomarkers in two consecutive harvest years emphasizes the importance of the metabolome study, for the traceability and quality control of the *V. album* preparations.

## 2. Results and Discussion

**Figure 1:**
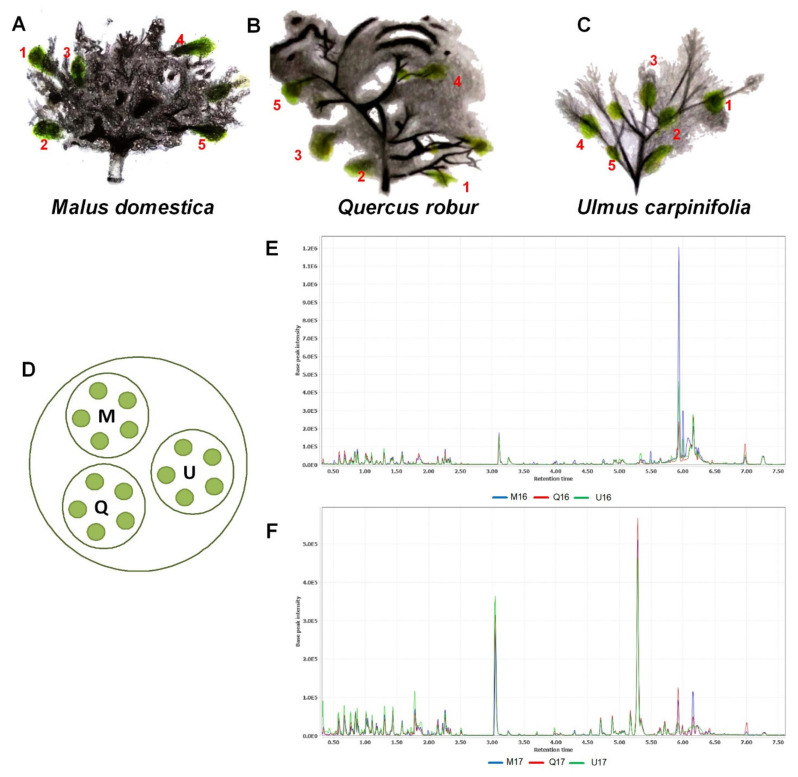
Five independent biological replicates from *V. album* ssp. *album* (1–5 in red) from the following different host trees: (**A**) *Malus domestica*, (**B**) *Quercus robur* and (**C**) *Ulmus carpinifolia*. (**D**) Harvest pattern of the *V. album* samples. Base peak chromatograms of the UHPLC-Q-TOF analysis conducted in 2016 (**E**) and 2017 (**F**). The codes represent the different host trees analyzed as follows: M (*M. domestica*); Q (*Q. robur*); U (*U. carpinifolia*).

Figure 1A–D shows a schematic drawing of the *V. album* ssp. *album*, harvested from each host tree (A: *M. domestica*; B: *Q. robur*; C: *U. carpinifolia*), located in the Canton Baselland (Switzerland). The metabolite fingerprinting was assessed by a UHPLC-TOF-MS^E^, using twenty-eight samples at the end of summer, in two subsequent years (2016, 2017). One of the five *M. domestica* samples harvested in 2016, and two technical replicates from 2017 were lost during the sampling process. The chromatographic profiles of the *V. album* samples were similar in each year of harvest, with only small differences in the peak intensities (Figure 1E,F).

After the preprocessing step, which led to 9800 markers and 7260 markers for the 2016 and 2017 batches, respectively, principal component analysis (PCA), an unsupervised analysis, was used to obtain a general overview of the possible clustering patterns. The PCA analysis showed three distinct clusters, in both the harvest years, with high differentiation among them (Figure S1).

**Figure 2:**
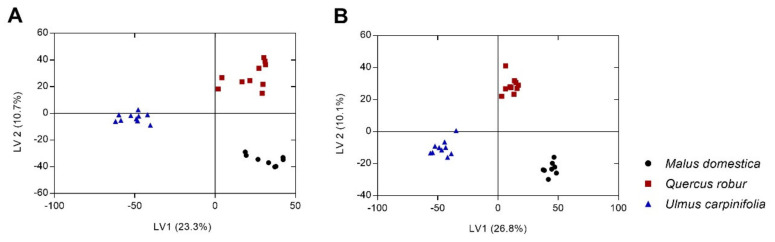
PLS-DA score plot for first and second latent variables showing the discrimination between *V. album* ssp. *album* from different host trees. Samples harvested in 2016 (**A**) and in 2017 (**B**). LV is latent variable.

In the second step, partial least squares discriminant analysis (PLS-DA), a supervised method, was conducted, to highlight the important variables in sample discrimination. Three clusters were observed, according to their following host trees: *V. album* ssp. *album* growing on *M. domestica*, *Q. robur*, and *U. carpinifolia* (Figure 2). The R^2^ and Q^2^ value coefficients were 95% and 88%, respectively, in the samples that were harvested in 2016, and 97% and 92%, respectively, in the samples that were collected in 2017, emphasizing the separation profiles among *V. album* ssp. *album* from different host trees, and a good fit and predictive ability with the PLS-DA model [19]. This was confirmed by permutation tests (*n* = 200), with R^2^ and Q^2^ intercepts at 0.771 and −0408 for the 2016 harvest, and 0.505 and −0.403 for the 2017 harvest. The PLS-DA scores plots (Figure 2A,B) showed that the latent variables LV1 and LV2 explained 23.3% and 10.7% (Figure 2A), and 26.8% and 10.1% of the data variability (Figure 2B), for the 2016 and 2017 harvests, respectively. Loadings plots are presented in Figure S2.

The data were clustered according to the host trees, contrary to the botanical classification that does not differentiate these samples, since all of them belong to the same subspecies, i.e., *V. album* ssp. *album*. These results highlight the differences in the chemical profiles among the samples of the host trees that were analyzed. The VIP score (variable importance in projection) was considered, to identify the features responsible for *V. album* spp. *album* differentiation. Table 1 summarizes the main characteristics of these high-VIP compounds, such as the following: the retention time, the experimental *m*/*z* value, the theoretical *m*/*z* value, the mass error, the neutral molecular formula, and the main deconvoluted MS/MS ions. Among the VIP generated by PLS-DA, 10 metabolites with a VIP score >5 were putatively identified, based on their MS/MS fragmentation pattern, compared to different MS/MS libraries and exact mass error <6 ppm, as shown in the Section 3.6.

In order to increase the chemical information, phenolic acids, flavonoids, amino acids, and others were putatively identified in the *V. album* ssp. *album* samples (Table 2). These compounds were also previously described in the *Viscum album* species [18,20,21,22,23]; however, they were not related to the differentiation of the three clusters that were described in the PLS-DA analysis.

**Figure 3:**
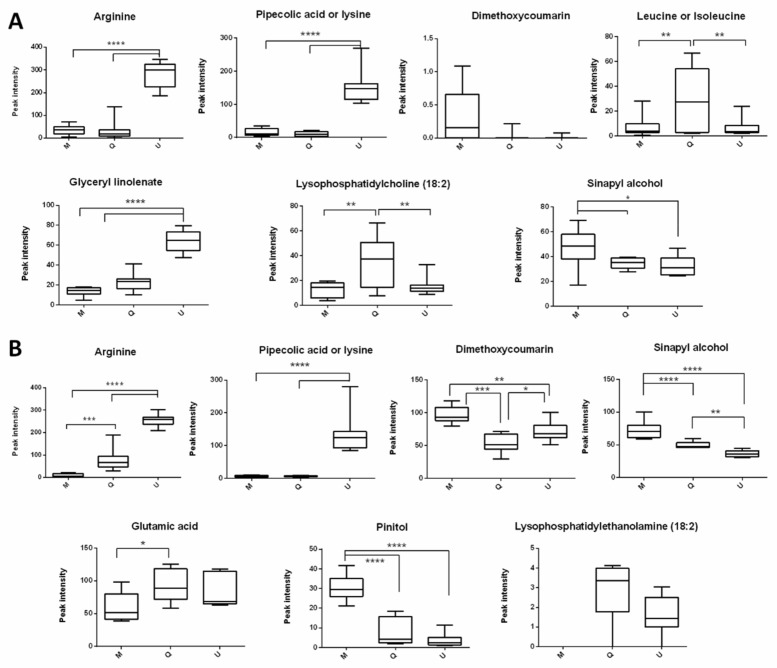
Intensity of the 2016 (**A**) and 2017 (**B**) VIP scores of *Viscum album* ssp. *album* from different host trees. M: *V. album* from *Malus domestica*, Q: *V. album* from *Quercus robur*, U: *V. album* from *Ulmus carpinifolia*.

High VIP score variables in PLS-DA strongly contribute to the samples differentiation [24]. In this work, amino acids, lipids, organic acids, and coumarin were included in the VIP-score shortlist, underlining their importance in discriminating *V. album* ssp. *album* groups. Each year revealed seven main compounds that were responsible for the *V. album*–host tree clustering, and four of them appeared in both the harvests (Table 1; Figure 3). In this analysis, arginine, pipecolic acid, or lysine presented a higher intensity in *V. album* ssp. *album* from *U. carpinifolia*. In addition, dimethoxycoumarin and sinapyl alcohol appeared to be predominant in *V. album* ssp. *album* from *M. domestica* (Figure 3A,B). The detected recurrence of these four compounds in both the years of summer harvest, as well as their intensities, suggest that they could be used as biomarkers of these raw materials. Therefore, our approach could be useful for the quality control of *V. album* ssp. *album* preparations. The other three VIP that were identified in 2016 (glyceryl linolenate, lysophosphatidylcholine, and leucine or isoleucine) and 2017 (glutamic acid, pinitol, and lysophosphatidylethanolamine) need to be further investigated, in order to reach any solid conclusions regarding their actual contributions as *V. album* biomarkers.

The quality control and standardization of plant extracts and herbal medicines include, among others, the source and quality of the raw materials, and good agricultural and manufacturing practices (GMP). The plants that were used in this work were harvested at the end of the European summer season (early September). The leaves, stems, and berries were collected from the same plants of *V. album*’s host trees, which were previously assigned by specific codes. GMP includes the establishment of growth conditions, harvesting, drying, and storage. Some elements, such as age, the part of the plant harvested, weather, time and method of collection, processing, and drying, can affect the quality, and thus the therapeutic activity, of the herbal medicines [25,26]. This is especially relevant for *V. album* ssp. *album*, since there are differences in biological activity, according to their host trees [17,27,28].

The use of *V. album* extracts for cancer complementary therapy is mainly standardized in concentrations of lectins and viscotoxins, which are high-molecular-weight compounds. However, the present work emphasizes the importance of small molecules, as well as the influence of the host tree on the metabolome of *V. album* ssp. *album*. The participation of these small molecules in the therapeutic potential of herbal preparations, should not be neglected, and needs further investigation. In Peñaloza and colleagues, we compared the metabolomic profile of fermented aqueous extracts from two different subspecies of *V. album*, i.e., ssp. *album* on *M. domestica* and ssp. *austriacum* on *Pinus sylvestris* [18]. We also registered a clear separation, according to the host tree species, by PLS-DA, and identified several primary and secondary metabolites, including amino acids, and organic and phenolic acids, emphasizing the importance of metabolome studies for the traceability of *V. album* preparations.

Arginine, a primary metabolite, with VIP score of 15.68, was the main compound that was responsible for the differentiation among the groups (Table 1), and presented high abundances in the *V. album* ssp. *album* from *U. carpinifolia* in both the years (Figure 3A,B). Arginine, and other amino acids, seem to be very important for the development of *V. album*, and have already been described in this species [29]. Urech previously reported arginine accumulation as an important form of nitrogen stock in *V. album* [30]. Zuber summed up *V. album* eco-physiological data and highlighted the importance of the high transpiration rates of this species, for its nutrition, also influencing the chemical composition of *V. album* [12]. This transpiration characteristic can be a parasite’s strategy to take up sufficient nitrogen from the host tree xylem, which is used to build proteins and other nitrogenous compounds that are important for the mistletoe development [31]. It is known that the content of nitrogen compounds in the host xylem depends on the nitrate nutrition and symbiotic N_2_ fixation. Since all the host trees evaluated in this work were not able to fix nitrogen [32], we suggest that the presence of arginine, as well as other amino acids that were identified in *V. album* ssp *album* from *U. carpinifolia*, could be explained by a difference in the nitrogenous soil nutrients around the host trees. New studies are needed to confirm this hypothesis, comparing the soil nutrient composition with the metabolome pattern of *V. album* host trees. 

Lysophosphatidylcholine (VIP 6.22; 2016) and lysophosphatidylethanolamine (VIP 5.88, 2017) were also involved in *V. album*–host tree differentiation (Table 1). Lipids are present in cell membranes, and changes in their composition can be related to internal and external stress. Welti and coworkers concluded that the freezing temperature was responsible for the variation in the *Arabidopsis* membrane lipid constitution [33]. The authors showed that, contrasting to plants growing at 19–23 °C, cold acclimation of *Arabidopsis* at 4 °C increased the polyunsaturated lysophospholipid species in the membrane, as a protective damage mechanism. This behavior indicates that membrane lipid composition has an important impact on freezing tolerance [34]. In our work, the harvests were carried out in the last month of European summer, when the temperatures were mild. In this sense, the accumulation of these phospholipids could be important to the maintenance of the membrane lipid bilayer structure, preparing the plants for the next season (autumn), when the temperature drops.

*V. album* is partially heterotrophic and acquires not only water and minerals from host trees, but also takes carbon from the xylem sap, to complement its own nutrition [12]. The chloroplasts of *V. album* present large deficiencies in their photosystems, which creates the need for a high carbon input from the host tree [12]. Senkler and colleagues showed that *Viscum album* have a lack of complex I in their mitochondrial oxidative phosphorylation (OXPHOS), which could explain its dependency on host tree metabolites [35]. In our study, the VIP scores showed pinitol as an important compound in cluster distinction, with a high intensity in *V. album* from *M. domestica*. It is well known that cyclitols are important sources of carbon compounds, establishing an osmotic balance and also acting as cryoprotectant in *V. album* [36].

In addition, other secondary metabolites were putatively identified in the present work. Dimethoxycoumarin presented higher abundances in *V. album* from *M. domestica* compared to the other host trees, and had the same signal intensity pattern in both the harvests. Coumarins, lactones of hydroxycinnamic acid, can be found glycosylated and in the free form [37]. These compounds are important in the ecological plant–plant interactions, because they act as allelochemicals, and delay the germination and growth of the same species or other plant species [37]. Additionally, the synthesis of dimethoxycoumarin could be intensified in *V. album* on *M. domestica*, for protection against fungal infection [38]. However, the relevance of these compounds in parasitic plants needs further studies. The possible direct uptake of the compounds, from the xylem sap of the host tree, might explain the host specific differences of *V. album*, but this still remains to be investigated.

One of the four compounds that was identified in all the *V. album* samples that were harvested, was sinapyl alcohol. This phenylpropanoid is a precursor of lignin or lignans, and of many stilbenes and coumarins [39]. Wagner and colleagues described some cardioactive phenylpropanes and lignans from *V. album* [40]. The authors demonstrated that sinapyl alcohol was a subunit of some lignans after phenylpropane aglycon acid hydrolysis. Lignins have important roles in the growth and development of plants. They are biopolymers that enhance plant cell wall rigidity and promote mineral transport. Moreover, lignin acts as a barrier, protecting plants against various external adverse factors, such as insect pests, diseases, salt, and temperature stress [41,42]. Wei and coworkers showed that the content of lignin in *Rhododendron* tissues was significantly increased in the process of cold acclimatization [43]. Furthermore, it is known that, in some plants, the deposition of lignin in seeds can protect them from external adverse factors, supporting propagation seed and species [42].

## 3. Materials and Methods

### 3.1. Chemicals and Reagents

Analytical grades solvents and reagents used for extraction were purchased from Sigma Aldrich (Darmstadt, Germany). LC-MS grade solvents were from Biosolve (Valkenswaard, The Netherlands). Purified water (B. Braun Melsungen AG, Melsungen, Germany) was used for *Viscum album* samples extraction.

### 3.2. Plant Growth and Harvest

The berries, leaves and stems of female mistletoe bushes were collected in September of 2016 and 2017 in the same location in Basel area (Höfli), close to GPS latitude and longitude coordinates 47.471351, 7.692720 in Switzerland. The climate at the site is temperate classified as Cfb by the Köppen–Geiger system, with annual average rainfall of 778 mm and temperature of 10.0 °C/51.1 °F [44]. *V. album* samples were harvested from five different bushes of the same angiosperm host tree, as follows: *M. domestica* (Rosaceae), *Q. robur* (Fagaceae), and *U. carpinifolia* (Ulmaceae), characterizing 5 biological replicates of each host tree. Since the plant metabolism is highly age-dependent and many metabolite levels are altered during the gradual plant ageing, the harvest standardization was conducted in order to collect plant material of the same level of development, regarding general morphological characteristics. For this work, two 1-year-old leaves and one 2-year-old leaf, one stem of each age (1- and 2-year-old), and three berries, were harvested on each mistletoe plant, as previously described by Holandino and coworkers [21]. The age of the *V. album* bush was estimated by counting the number of nodes on the longest branch of mistletoe. All collections were conducted in the morning (between 8:00 and 11:30 am), and the thirty samples were immediately frozen in liquid nitrogen.

### 3.3. Plant Extraction

The frozen plant material, containing a pool of leaves, berries, and stems, was manually ground to a fine powder in a pre-cooled mortar and pestle, under liquid nitrogen, following methodology previously established [12]. Approximately 150 mg of powder (145–170 mg) was transferred to a 1.5 mL microcentrifuge tube under frozen conditions. One mL of methanol–formic acid–water (80:20:0.5, *v*/*v*) as well as 5–10 glass beads (1.0–1.5 mm diameter) were added and the mixture was homogenized for 3 min, at 30 Hz using a bead mill (Retsch MM 400, Haan, Germany). Technical duplicates were centrifuged (5 min, 14,000× *g*) and the supernatants were filtered through 13 mm PTFE syringe filters (0.22 µm pore size, BGB, Alexandria, VA, USA), immediately before the analysis. In addition, quality control samples (QC) were prepared by pooling 20 µL of all samples. Samples were kept at 4 °C until analysis.

### 3.4. UHPLC-TOF-MS Conditions

Metabolomics analyses were performed on an Acquity UPLC system coupled to a Synapt G2 Q-TOF mass spectrometer (Waters, Milford, MA, USA) using conditions adapted from Gaillard and coworkers [45]. The column used for separation was an Acquity UPLC BEH C18 (Waters) and the mobile phases were (A) H_2_O + formic acid 0.05% and (B) acetonitrile + formic acid 0.05%. The following gradient program was used at a flow rate of 0.6 mL/min and a temperature of 40 °C: 2–100% B in 6.0 min, hold at 100% for 1.5 min, and at 2% B for 1.5 min. The injection volume was 1 μL. The high-resolution mass spectrometer was operated in positive electrospray ionization using the so-called MS^E^ mode over a mass range of 85–1200 Da. MS^E^ is a data-independent acquisition mode, which records data without preselection of parent ions by alternatively switching from low to high collision energies. The following source conditions were used: capillary voltage +2800 V, cone voltage +25 V, source temperature 120 °C, desolvation temperature 400 °C, desolvation gas flow 900 L/h, and cone gas flow 20 L/h. Data were acquired in centroid mode at a resolution of ca. 20,000 (at *m*/*z* 556). Scan time was set to 0.15 s, allowing more than 10 data points across chromatographic peaks. Internal calibration was performed through the Lockspray interface (Waters) by infusing a 500 ng/mL solution of leucine-enkephalin in the mass spectrometer at a flowrate of 15 μL/min. The system was controlled by Masslynx 4.1 (Waters).

### 3.5. UHPLC-TOF-MS Data Processing and PLS-DA Analysis

Peak picking was carried out in Markerlynx XS (Waters) using the following parameters: retention time window, 0.0–5.75 min; mass range, 85–1200 Da; mass window, 0.02 Da; retention time window, 0.06 min; intensity threshold, 500 counts; automatic peak width and peak-to-peak baseline noise calculation, deisotoping applied. The obtained peak lists made of observations (i.e., samples) in columns and variables (i.e., markers of given retention time and *m*/*z*) in lines, were exported to SIMCA software (v. 13) for principal component analysis (PCA) and partial least squares discriminant analysis (PLS-DA). Data were Pareto-scaled prior to multivariate analysis. Since PLS-DA can be prone to overfitting, all PLS-DAs models were validated using leave-one-subject-out cross-validation with R^2^ and Q^2^ metrics and permutations tests (*n* = 200).

### 3.6. Metabolite Annotation

Raw data files were converted to ABF format using the ABF converter software (https://www.reifycs.com/AbfConverter/ accessed on 21 April 2019). Then, ABF files were submitted to peak picking, alignment, deconvolution, and identification processes using the freely available MS-DIAL software (v 3.40) [46]. The parameters used in MS-DIAL were as follows: MS1 and MS2 tolerances 0.01 and 0.05, respectively; minimum peak height of 1000; mass slice width of 0.05 Da; linear-weighted moving average as the smoothing method using 3 scans and peak width of 5 scans; sigma window value for deconvolution of 0.4; 0.1 min and 0.01 Da tolerance for peak alignment. Compound annotation was performed by comparing the aligned *m*/*z* ions and their deconvoluted MS/MS spectra to those uploaded to the MassBank of North America (http://mona.fiehnlab.ucdavis.edu/ accessed on 17 June 2019) and the NIST 2014 MS/MS library.

## 4. Conclusions

The present results provide new insights into the chemical composition of *V. album* ssp. *album* from different deciduous host trees. Untargeted UHPLC-QTOF-MS, combined with PLS-DA, clustered samples into three groups according to the three distinct host trees and putatively identified *V. album* small molecules that are responsible for this differentiation, consisting of amino acids, lipids, organic acids, and coumarin. Arginine, pipecolic acid, or lysine proved to be important biomarkers of *V. album* ssp. *album* from *U. carpinifolia*, and dimethoxycoumarin and sinapyl alcohol appeared as chemical markers of *V. album* ssp. *album* from *M. domestica*. These four identified VIPs were the same in both years of harvest and presented similar abundances in the three host species that were investigated, showing extraction reproducibility, as well as a stability of the metabolites extracted, highlighting the importance of the harvest standardization procedure to biomarkers identification. These aspects have been reflected in the reproducibility of the present results, and also emphasize the importance of the metabolome study for the traceability and quality control of the *V. album* preparations.

## Figures and Tables

**Table 1:** Discriminant compounds of the *V. album* ssp. *album* groups obtained by PLS-DA model.

VIP Score 2016	VIP Score 2017	t_R_ (min)	*m*/*z* Theoretical *[M + H]^+^*	Error (ppm)	Neutral Formula	Compounds	Deconvoluted MS/MS Ions
15.68	15.68	0.24	175.11895	3.71	C_6_H_14_N_4_O_2_	Arginine	175.12; 158.09; 116.07
12.8	12.8	0.33	130.08626	1.84	C_6_H_11_NO_2_	Pipecolic acid or lysine	130.09; 84.05; 56.05
9.03	9.03	1.31	207.06519	1.50	C_11_H_10_O_4_	Dimethoxycoumarin	207.07; 175.04; 147.04; 119.05
7.97	- *	0.47	132.10191	2.95	C_6_H_13_NO_2_	Leucine or Isoleucine	132.1; 86.1; 69.07
6.68	- *	4.01	353.26864	0.17	C_21_H_36_O_4_	Glyceryl linolenate	353.27; 335.26; 317.25; 279.23; 261.22; 243.21
6.22	- *	3.98	520.33977	−1.48	C_26_H_50_NO_7_P	LysoPC (18:2) ^a^	520.34; 502.32; 184.07; 86.09
5.94	5.94	1.03	193.08592	4.56	C_11_H_14_O_4_	Sinapyl alcohol	193.08; 161.06;133.06;105.07
- *	5.93	0.26	148.06043	5.88	C_5_H_9_NO_4_	Glutamic acid	148.06; 130.05; 102.05
- *	5.90	0.25	195.08631	4.56	C_7_H_14_O_6_	Pinitol	127.04; 109.03; 85.03; 81.03; 71.05
- *	5.88	3.96	478.29282	−1.09	C_23_H_44_NO_7_P	LysoPE (18:2) ^b^	337.27; 109.1; 95.08; 64.04

*—not detectable as a VIP > 5 in this year; ^a^ lysophosphatidylcholine (18:2); ^b^ lysophosphatidyl-ethanolamine (18:2).

**Table 2:** Other *V. album* ssp. *album* compounds putatively identified.

t_R_ (min)	*m*/*z*	*m*/*z* Theoretical *[M + H]^+^*	Error (ppm)	Neutral Formula	Compounds	Deconvoluted MS/MS Ions
0.30	127.0395	127.03897	4.17	C_6_H_6_O_3_	Phloroglucinol	127.0394; 109.0117; 81.0085; 68.995; 53.0412
0.35	308.0915	308.09108	1.36	C_10_H_17_N_3_O_6_S	Glutathione (reduced)	308.0904; 179.0486; 162.0231; 84.0456; 76.022
0.40	182.0814	182.08117	1.26	C_9_H_11_NO_3_	Tyrosine	182.0816; 165.056; 136.0571; 123.0463; 119.0497; 91.0548
0.85	355.1035	355.10236	3.21	C_16_H_18_O_9_	Chlorogenic acid	355.1042; 163.0818; 145.0045; 135.0049; 117.0762; 107.0501
0.89	235.1452	235.1441	4.68	C_13_H_18_N_2_O_2_	Coumaroyl putrescin	234.1451; 147.0448; 119.0503
1.04	390.176	390.17586	0.36	C_17_H_24_O_9_	Syringin	193.087; 161.06505, 166.0655; 105.0706
1.50	465.1032	465.10275	0.97	C_21_H_20_O_12_	Quercetin-*O*-glucoside	465.1021; 303.0875
1.69	435.128	435.12857	−1.31	C_21_H_22_O_10_	Naringenin-*O*-glucoside	435.1292; 273.0391
1.80	301.1084	301.10705	4.48	C_17_H_16_O_5_	Flavanone ^a^	301.1098; 181.0619
4.43	522.3555	522.35542	0.15	C_26_H_52_NO_7_P	LysoPC (18:1) ^b^	522.3539; 184.0736; 104.1069
5.67	758.5683	758.56943	1.49	C_42_H_80_NO_8_P	PC (16:0/18:2) ^c^	758.5747; 184.0746

^a^ Dimethoxy hydroxyflavanone; ^b^ lysophosphatidylcholine (18:1); ^c^ phosphatidylcholine (16:0/18:2).

## Supplementary Materials

The following are available online at https://www.mdpi.com/article/10.3390/plants10081726/s1, Figure S1: PCA score plot for first and second principal components (PC) showing the discrimination between *V. album* ssp. *album* from different host trees was included. Samples harvested in 2016 (**A**) and in 2017 (**B**), Figure S2: PLS-DA loadings plots for 2016 (**A**) and 2017 (**B**).

## References

[1] Gupta G., Kazmi I., Afzal M., Rahman M., Saleem S., Ashraf M.S., Khusroo M.J., Nazeer K., Ahmed S., Mujeeb M. (2012). Sedative, antiepileptic and antipsychotic effects of *Malus album* L. (Loranthaceae) in mice and rats. J. Ethnopharmacol..

[2] Suveren E., Baxter G.F., Iskit A.B., Turker A.U. (2017). Cardioprotective effects of *Viscum album* L. subsp. *album* (European misletoe) leaf extracts in myocardial ischemia and reperfusion. J. Ethnopharmacol..

[3] Ramm H. (2015). Mistletoe through Cultural and Medical History: The All-Healing Plant Proves to Be a Cancer-Specific Remedy. Transl. Res. Biomed..

[4] Song C., Wei X.Y., Qiu Z.D., Gong L., Chen Z.Y., Ma Y., Shen Y., Zhao Y.J., Wang W.h., Lai C.J.S. (2021). Exploring the resources of the genus *Viscum* for potential therapeutic applications. J. Ethnopharmacol..

[5] Ostermann T., Appelbaum S., Poier D., Boehm K., Raak C., Bussing A. (2020). A Systematic Review and Meta-Analysis on the Survival of Cancer Patients Treated with a Fermented *Viscum album* L. Extract (Iscador): An Update of Findings. Complement. Med. Res..

[6] Loef M., Walach H. (2020). Quality of life in cancer patients treated with mistletoe: A systematic review and meta-analysis. BMC Complement. Med. Ther..

[7] Hegde P., Maddur M.S., Friboulet A., Bayry J., Kaveri S.V. (2011). *Viscum album* exerts anti-inflammatory effect by selectively inhibiting cytokine-induced expression of cyclooxygenase-2. PLoS ONE.

[8] Abdel-Salam O.M., Sleem A.A., Shaffie N.M. (2010). Effect of *Viscum album* on acute hepatic damage caused by carbon tetrachloride in rats. Turkish J. Med. Sci..

[9] Ahmed A.K., Mert N. (2019). Investigation of the Antidiabetic Effects of Mistletoe (*Viscum album* L.) Extract in Experimental Diabetes in Rats. Van Vet. J..

[10] Orhan D.D., Aslan M., Sendogdu N., Ergun F., Yesilada E. (2005). Evaluation of the hypoglycemic effect and antioxidant activity of three *Viscum album* subspecies (European mistletoe) in streptozotocin-diabetic rats. J. Ethnopharmacol..

[11] Ertürk Ö. (2003). Antimicrobial Activity of *Viscum album* L. subsp. *abietis* (Wiesb). Turkish J. Biol..

[12] Zuber D. (2004). Biological flora of Central Europe: *Viscum album* L.. Flora.

[13] Jäger T., Holandino C., Glauser G., Grazi M., Ramm H., de Oliveira Melo M.N., Oliveira A.P., Garrett R., Baumgartner S. (2019). Metabolic profiling as a tool for differentiating *Viscum album* ssp. *album* plants growing on various host trees. Phytomedicine.

[14] Vicaş S.I., RuginǍ D., Leopold L., Pintea A., Socaciu C. (2011). HPLC Fingerprint of bioactive compounds and antioxidant activities of *Viscum album* from different host trees. Not. Bot. Horti Agrobot..

[15] Farag M.A., Westphal H., Eissa T.F., Wessjohann L.A., Meyer A. (2017). Effect of oxylipins, terpenoid precursors and wounding on soft corals’ secondary metabolism as analyzed via UPLC/MS and chemometrics. Molecules.

[16] Walker J.M. (2013). Metabolomics Tools for Natural Product Discovery.

[17] Zhang R.-Z., Zhao J.-T., Wang W.-Q., Fan R.-H., Rong R., Yu Z.-G., Zhao Y.-L. (2021). Metabolomics-based Comparative Analysis of the Effects of Host and Environment on *Viscum coloratum* Metabolites and Antioxidative Activities. J. Pharm. Anal..

[18] Peñaloza E., Holandino C., Scherr C., de Araujo P.I.P., Borges R.M., Urech K., Baumgartner S., Garrett R. (2020). Comprehensive Metabolome Analysis of Fermented Aqueous Extracts of *Viscum album* L. By Liquid Chromatography−High Resolution Tandem Mass Spectrometry. Molecules.

[19] Pérez-Míguez R., Sánchez-López E., Plaza M., Castro-Puyana M., Marina M.L. (2018). A non-targeted metabolomic approach based on reversed-phase liquid chromatography–mass spectrometry to evaluate coffee roasting process. Anal. Bioanal. Chem..

[20] Melo M.N.d.O., Oliveira A.P., Wiecikowski A.F., Carvalho R.S., Castro J.d.L., de Oliveira F.A.G., Pereira H.M.G., da Veiga V.F., Capella M.M.A., Rocha L. (2018). Phenolic compounds from *Viscum album* tinctures enhanced antitumor activity in melanoma murine cancer cells. Saudi Pharm. J. SPJ Off. Publ. Saudi Pharm. Soc..

[21] Holandino C., Melo M.N.d.O., Oliveira A.P., Batista J.V.d.C., Capella M.A.M., Garrett R., Grazi M., Ramm H., Torre C.D., Schaller G. (2020). Phytochemical analysis and in vitro anti-proliferative activity of *Viscum album* ethanolic extracts. BMC Complement. Med. Ther..

[22] Szurpnicka A., Zjawiony J.K., Szterk A. (2019). Therapeutic potential of mistletoe in CNS-related neurological disorders and the chemical composition of *Viscum* species. J. Ethnopharmacol..

[23] Pietrzak W., Nowak R., Gawlik-Dziki U., Lemieszek M.K., Rzeski W. (2017). LC-ESI-MS/MS Identification of Biologically Active Phenolic Compounds in Mistletoe Berry Extracts from Different Host Trees. Molecules.

[24] Fernandez O., Urrutia M., Berton T., Bernillon S., Deborde C., Jacob D., Maucourt M., Maury P., Duruflé H., Gibon Y. (2019). Metabolomic characterization of sunflower leaf allows discriminating genotype groups or stress levels with a minimal set of metabolic markers. Metabolomics.

[25] Kunle O.F., Egharevba H.O., Ahmadu P.O. (2012). Standardization of herbal medicines—A review. Int. J. Biodivers. Conserv..

[26] WHO (2018). Annex 1: WHO Guidelines on Good Herbal Processing Practices for Herbal Medicines.

[27] Urech K., Giannattasio M., Schaller G., Urech K. (1996). Cytotoxicity of Different Viscotoxins and Extracts from the European Subspecies of *Viscum album* L.. Phytotherapy Res..

[28] Bonamin L.V., Carvalho A.C.D.E., Waisse S. (2017). *Viscum album* (L.) in experimental animal tumors: A meta-analysis. Exp. Ther. Med..

[29] Escher P., Eiblmeier M., Hetzger I., Rennenberg H. (2004). Spatial and seasonal variation in amino compounds in the xylem sap of a mistletoe (*Viscum album*) and its hosts (*Populus* spp. and *Abies alba*). Tree Physiol..

[30] Urech K. (1997). Accumulation of arginine in *Viscum album* L.: Seasonal variations and host dependency. J. Plant Physiol..

[31] Schulze E.-D., Turner N.C., Glatzel G. (1984). Carbon, water and nutrient relations of two mistletoes and their hosts: A hypothesis. Plant. Cell Environ..

[32] Newton W.E. (2007). Physiology, Biochemistry, and Molecular Biology of Nitrogen Fixation. Biology of the Nitrogen Cycle.

[33] Welti R., Li W., Li M., Sang Y., Biesiada H., Zhou H.E., Rajashekar C.B., Williams T.D., Wang X. (2002). Profiling membrane lipids in plant stress responses: Role of phospholipase Dα in freezing-induced lipid changes in arabidopsis. J. Biol. Chem..

[34] Steponkus P.L. (1984). Injury and Cold Acclimation. Annu. Rev. Plant Physiol..

[35] Senkler J., Rugen N., Eubel H., Hegermann J., Braun H.P. (2018). Absence of Complex I Implicates Rearrangement of the Respiratory Chain in European Mistletoe. Curr. Biol..

[36] Richter A., Popp M. (1992). The physiological importance of accumulation of cyclitols in *Viscum album* L.. New Phytol..

[37] Kupidlowska E., Dobrzynska K., Parys E., Zobel A.M. (1994). Effect of coumarin and xanthotoxin on mitochondrial structure, oxygen uptake, and succinate dehydrogenase activity in onion root cells. J. Chem. Ecol..

[38] Sulistyowati L., Keane P.J., Anderson J.W. (1990). Accumulation of the phytoalexin, 6,7-dimethoxycoumarin, in roots and stems of citrus seedlings following inoculation with *Phytophthora citrophthora*. Physiol. Mol. Plant Pathol..

[39] Higuchi T. (1990). Lignin biochemistry: Biosynthesis and biodegradation. Wood Sci. Technol..

[40] Wagner H., Jordan E., Feil B. (1986). Studies on the standardization of mistletoe preparations. Oncology.

[41] Moura J.C.M.S., Bonine C.A.V., de Oliveira Fernandes Viana J., Dornelas M.C., Mazzafera P. (2010). Abiotic and biotic stresses and changes in the lignin content and composition in plants. J. Integr. Plant Biol..

[42] Liu Q., Luo L., Zheng L. (2018). Lignins: Biosynthesis and biological functions in plants. Int. J. Mol. Sci..

[43] Wei H., Dhanaraj A.L., Arora R., Rowland L.J., Fu Y., Sun L. (2006). Identification of cold acclimation-responsive Rhododendron genes for lipid metabolism, membrane transport and lignin biosynthesis: Importance of moderately abundant ESTs in genomic studies. Plant Cell Environ..

[44] Climate-Data.org. https://en.climate-data.org/europe/switzerland/basel-city/basel-437/.

[45] Gaillard M.D.P., Glauser G., Robert C.A.M., Turlings T.C.J. (2018). Fine-tuning the ‘plant domestication-reduced defense’ hypothesis: Specialist vs. generalist herbivores. New Phytol..

[46] Tsugawa H., Cajka T., Kind T., Ma Y., Higgins B., Ikeda K., Kanazawa M., Vandergheynst J., Fiehn O., Arita M. (2015). MS-DIAL: Data-independent MS/MS deconvolution for comprehensive metabolome analysis. Nat. Methods.

