# Supplementary material for: Metabolomics by UHPLC-Q-TOF Reveals Host Tree-Dependent Phytochemical Variation in Viscum album L
Source: Plants (Basel). 2021 Aug 20;10(8):1726. doi: 10.3390/plants10081726 (PMC8399568; doi:10.3390/plants10081726)
Supplement: Supplementary file 1 [file plants-10-01726-s001.zip › plants-1315229-supplementary.pdf]

Supplementary Material

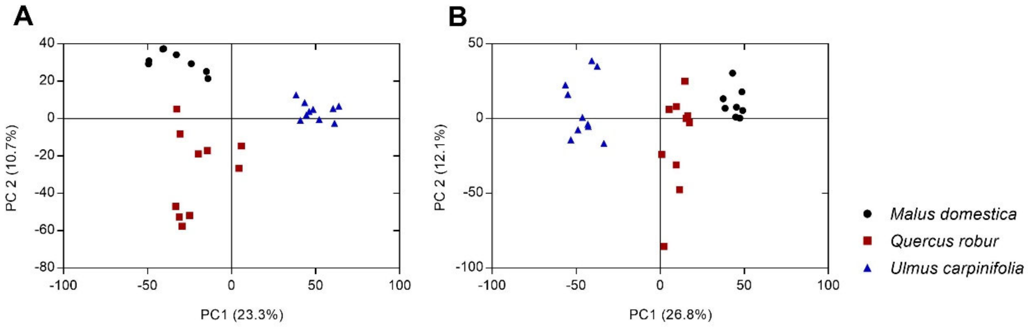

Figure S1. PCA score plot for first and second principal components (PC) showing the discrimination between *V. album* ssp. *album* from different host trees. Samples harvested in 2016 (**A**) and in 2017 (**B**).

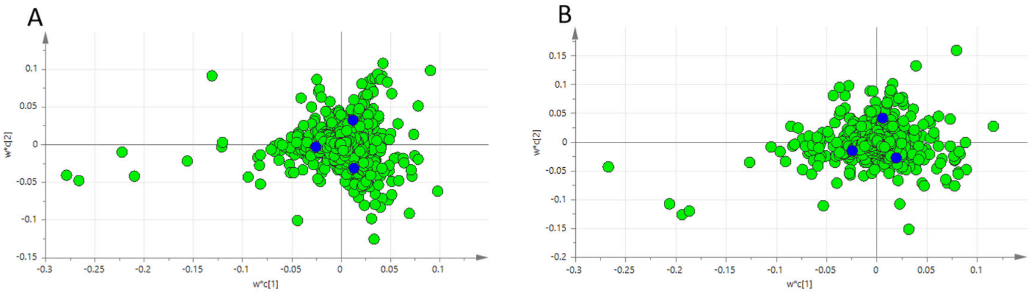

Figure S2. PLS-DA loadings plots for 2016 (**A**) and 2017 (**B**).
